# Supplementary figures and images for: Sex ratio and relatedness in the Griffon vulture (Gyps fulvus) population of Serbia
Source: PeerJ. 2022 Dec 7;10:e14477. doi: 10.7717/peerj.14477 (PMC9745909; doi:10.7717/peerj.14477)

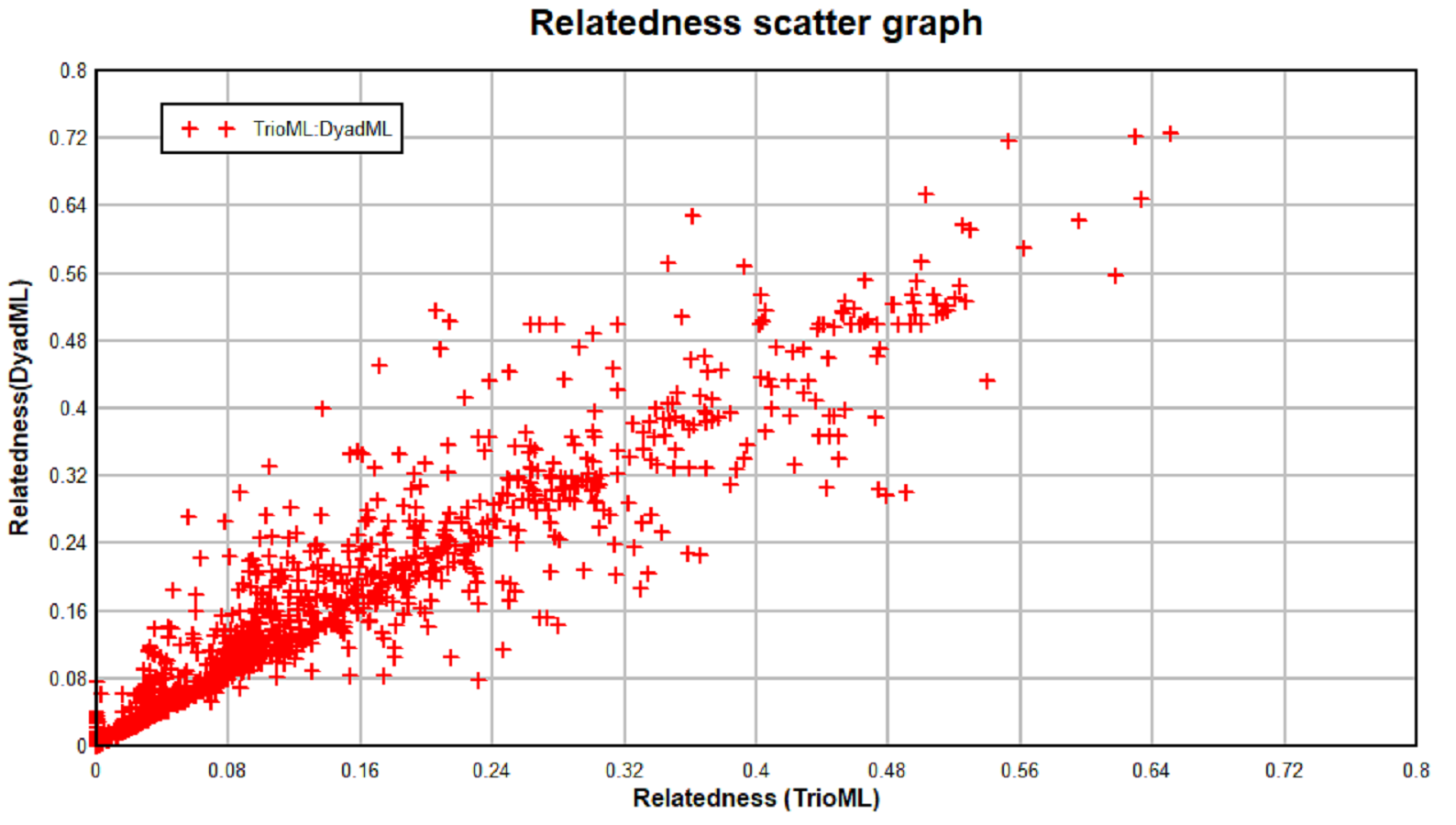

Supplement: Supplemental Information 1 — Scatter graph represents correlation between the TrioML and DyadML estimators for corresponding individual dyads. Relatedness estimates used for plotting TrioML and DyadML estimators in scatter graph are listed in Table S3. [file peerj-10-14477-s001.png]
